# Cost Disparities with Age in the Treatment of Advanced Non-Small-Cell Lung Cancer (NSCLC) in Ontario, Canada

**DOI:** 10.3390/curroncol32060346

**Published:** 2025-06-12

**Authors:** Ying Wang, Greg Pond, Amiram Jacob Gafni, Chung Yin Kong, Peter M. Ellis

**Affiliations:** 1 Department of Oncology, BC Cancer Vancouver, University of British Columbia; Vancouver, BC V5Z 4E6, Canada; 2 Ontario Clinical Oncology Group, Department of Oncology, Juravinski Hospital and Cancer Centre, McMaster University, Hamilton, ON L8V 1C3, Canada; gpond@mcmaster.ca; 3 Department of Health Research Methods, Evidence and Impact, Centre for Health Economics and Policy Analysis, McMaster University, Hamilton, ON L8S 4L8, Canada; gafni@mcmaster.ca; 4 Department of Medicine, Icahn School of Medicine at Mount Sinai, New York, NY 10029, USA; chungyin.kong@mountsinai.org; 5 Department of Oncology, Juravinski Hospital and Cancer Centre, McMaster University, Hamilton, ON L8V 1C3, Canada

**Keywords:** advanced NSCLC, phases of care, age, cost analysis, chemotherapy

## Abstract

Previous studies have noted associations between age and healthcare costs in non-small-cell lung cancer (NSCLC). However, the drivers of cost disparities have not yet been fully examined. This retrospective cohort study included deceased patients diagnosed with stage IV NSCLC in Ontario from 1 April 2008 to 30 March 2014. Variables of interest were extracted from the Institute for Clinical Evaluative Sciences. Average monthly cancer-attributable costs (CACs), defined as the net additional costs due to cancer, determined by subtracting pre-diagnosis costs from post-diagnosis costs, were calculated by phases of care (staging, initial, continuing, and end-of-life). Regression analyses assessed predictors of cost variability. The median age of the 14,655 patients was 65 to 69 years; 54% were male and 29% had received chemotherapy. On both univariate and multivariate analysis, CACs decreased with age after cancer diagnosis across all phases of care (*p* < 0.001). Receiving chemotherapy contributed to higher costs in staging, initial, and continuing phases (OR 2.11, 95% C.I. 1.90–2.33, *p* < 0.01), and lower costs in the end-of-life phase (OR 0.77, 95% C.I. 0.72–0.81, *p* < 0.01). Our study showed that older patients had higher baseline healthcare costs and lower cancer-attributable costs following diagnosis of advanced NSCLC. Cost drivers, including treatment and gender, varied by phase of care.

## 1. Introduction

Lung cancer is mainly a disease of the elderly, with a median age at diagnosis of 70 years [1,2]. As this population represents most lung cancer cases, understanding the costs associated with care, such as hospitalization and chemotherapy use, is important for effective healthcare planning. Previous studies have identified that elderly patients are both less likely to receive guideline recommended treatment, and less likely to receive therapies at all [1,2,3,4]. However, increased comorbidities and a relatively lower functional reserve may lead to increased adverse events, and potential additional costs to the healthcare system [2]. Despite these challenges, the specific costs associated with treating elderly lung cancer patients, especially in a publicly funded healthcare system, require further characterization.

Several retrospective cost analyses from different countries have been published examining age-related cost of care for advanced and, in particular, stage IV non-small-cell lung cancer (NSCLC). For example, amongst those 65 years or older, the costs of cancer care seem to decrease with age amongst the US Surveillance, Epidemiology and End Results (SEER)-Medicare database. Similar findings were seen amongst the British patients with advanced NSCLC [5,6,7]. Currently, there remains a knowledge deficit in both the total cost of treating patients as a function of age, including those younger than 65, as well as whether drivers of costs differ across age groups. These are important factors to consider, especially with anticipated changes in population demographics and the introduction of costlier therapies. Thus, it is important to analyze historical trends in costs and treatments by age to accurately assess and predict costs for future NSCLC care.

The American Society for Clinical Oncology (ASCO) has emphasized concerns regarding the escalating cost of cancer care and has acknowledged cost-effectiveness as an additional factor when considering treatment guidelines [8]. We were interested in examining the real-world costs of advanced NSCLC over the last two decades in Ontario. We conducted a study assessing the healthcare utilization rate, and the total and cancer-attributable costs (CACs) on a population level. Secondly, we hoped to use this as a model of care to predict factors associated with higher costs of care and help anticipate future healthcare spending needs of lung-cancer care as new treatments are introduced.

## 2. Materials and Methods

We conducted a retrospective, population-based cohort study on resource utilization and cost of care for patients diagnosed between 1 April 2008 to 30 March 2014 with follow-up through to 30 March 2017, who had passed away with stage IV NSCLC, and who had been treated within any one of fourteen healthcare regions within Ontario, Canada, for whom health utilization information was available. This study specifically focused on estimating cancer-attributable costs (CACs), defined as the net additional healthcare costs incurred by a cancer diagnosis, by subtracting each patient’s baseline pre-diagnosis costs from their post-diagnosis costs.

### 2.1. Institute for Clinical Evaluative Sciences (IC-ES) Data

The available provincial administrative databases facilitate the evaluation of the utilization of services and cost of care for all patients eligible for provincial healthcare. Health services research within Ontario, the largest province in Canada by population, is facilitated through the amalgamation of data from multiple provincial databases managed by the Institute for Clinical Evaluative Sciences (IC-ES), which allows for pooled data analysis of treatment patterns for cancer care across the province. Provincial databases include the Ontario Cancer Registry, the Cancer Activity Level Reporting, and the New Drug Funding Program within Ontario Health—Cancer Care Ontario, and databases such as the National Ambulatory Care Reporting System and the Continuing Care Reporting System under the Canadian Institute for Health Information. Individual patient data can then be linked via data from the Ontario Health Insurance Plan (OHIP) and the Ontario Drug Benefit Claims database, which provides medication coverage for seniors (people 65 years and older) and those younger receiving benefits from social assistance [9]. OHIP is Ontario’s publicly funded health insurance program that covers Ontario residents for health services including outpatient physician visits, medical tests such as laboratory tests, and procedures such as surgeries and radiation therapy. This allows assessment of both practice patterns and cost analysis for the entire population, as well as by individual healthcare regions, and in rural versus urban settings. This study received approval from the Hamilton Integrated Research Ethics Board (Project ID 4191), ensuring compliance with ethical standards for analysis of healthcare data.

Mean monthly cost of care was calculated, including costs of systemic therapy and supportive care (hospitalization, physician billings, lab tests, emergency visits, home care, and most prescription medications). Payments were converted to constant 2015 CAD by fiscal year of diagnosis. For example, fiscal year 2013 extends from 1 April 2013 to 31 March 2014, inclusively. The use of oral systemic therapy is not universally captured in these databases, as prescription medications are only fully captured in patients who are 65 years of age or older and among patients enrolled in government assistance programs.

As per IC-ES privacy rules, age was stratified into the following groups: <55, 55–59, 60–64, 65–69, 70–74, 76–79, and ≥80 years of age. Covariates of interest included gender; Charlson comorbidities index (CCI); year of diagnosis; rural versus urban living; and receipt of systemic therapy, largely intravenous chemotherapy, versus no systemic therapy received. Radiation usage was measured through billing data.

### 2.2. Phases of Care

**Figure 1:**
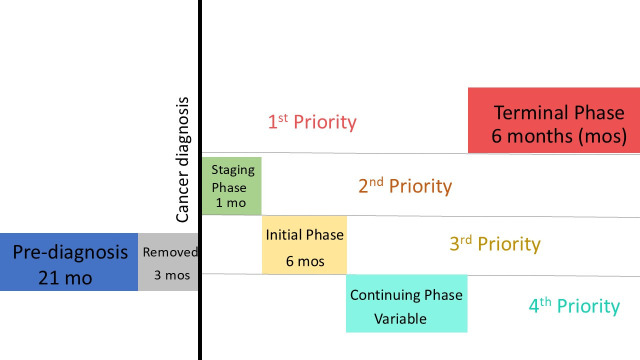
Visual framework for calculating phases-of-care.

Costs were calculated according to phases of care, based on the cost methodology developed and implemented by Sheehan et al. for the SEER database assessing Medicare data, which shares a similar structure to Ontario’s OHIP funding (Figure 1) [6]. The pre-diagnosis phase was defined as the 21-month period ending three months prior to the diagnosis of NSCLC. The pre-diagnosis phase was included to provide an accurate baseline of patients’ healthcare costs prior to NSCLC diagnosis. The three months prior to the diagnosis were excluded from the pre-diagnosis phase cost calculations to avoid potential confounding costs from healthcare costs incurred during work-up of NSCLC histological diagnosis.

The post-diagnosis period was split into four phases of care: staging, initial, continuing, and end-of-life (or terminal) phases. Given the expected median survival of advanced NSCLC patients, the end-of-life phase took priority and was defined as the last six months of the patient’s life, ending on the date of death. The duration of the end-of-life phase was previously defined by Sheehan et al., who demonstrated that costs of NSCLC care increased around 6 months prior to death [6]. The staging phase was defined as the first month after diagnosis and not falling within the end-of-life phase, followed by an initial phase that lasted up to six months immediately following the staging phase and, again, not falling within the end-of-life phase. The continuing phase varied in length depending on how long patients lived after diagnosis of cancer. For example, a patient who lived 14 months after diagnosis of cancer would contribute 6 months towards the end-of-life phase, 1 month to the staging phase, 6 months to the initial phase, and 1 month to the continuing phase. These phase-specific costs were then used to calculate the CACs across distinct intervals of the cancer-care trajectory.

### 2.3. Cancer-Attributable Costs

CACs were calculated by the net cost method, in which each patient’s post-diagnosis healthcare costs are subtracted from their own pre-diagnosis baseline costs, as measured in the 18 month period from 21 to 3 months prior to their diagnosis [7]. This approach isolated the net costs attributable specifically to cancer-related care, adjusting for individual variation in comorbidities and baseline healthcare use. All costs were provided by healthcare utilization categories by IC-ES. Calculating the CACs allowed for control for certain variables within IC-ES, such as each patient’s individual comorbid conditions and healthcare utilization habits [6]. 

### 2.4. Statistical Analysis

Descriptive analysis was conducted to assess the rate of utilization of systemic therapy at a population level and by age group. Survival estimates were calculated using the Kaplan–Meier method. Comparative and frequency statistics were used to describe the utilization pattern for each population in question. Cost analysis was conducted based on aggregate cost of care and CACs for the health system. Separate models were fitted for each phase of care. Univariate linear regression analysis was used to estimate association of age with the log-transformed cost of care. Multivariate linear regression analysis was used to assess the relationship between log-transformed CACs of care in each phase of care with age, gender, year of diagnosis, income quintile, rural vs. urban living, and whether systemic therapy was delivered. The adjusted relative costs were obtained by back-transformation to the linear scale. All costs were accounted for from a public-payer-system perspective. Analysis was performed using STATA 17.0 (StataCorp. 2021. College Station, TX, USA: StataCorp LLC.).

## 3. Results

### 3.1. Baseline Demographics

**Figure 2:**
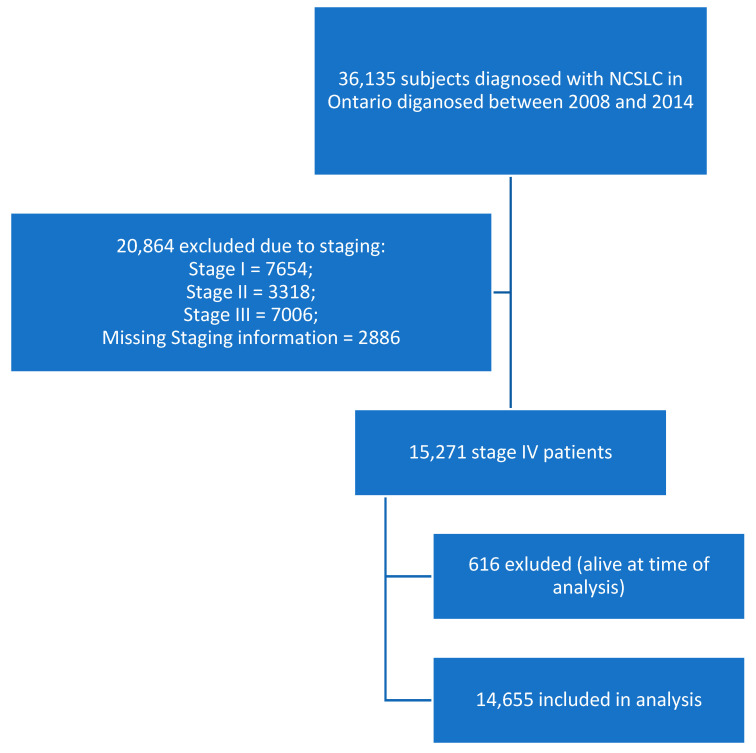
Consort diagram.

A total of 36,135 patients were diagnosed with NSCLC in Ontario between 2008 and 2014 (Figure 2). Of those, 15,271 patients had documented stage IV disease at diagnosis and 616 patients were still alive. Thus, 14,655 patients were included in this Ontario cohort. Table 1 shows the baseline demographics of patients across age groups. Notably, the proportion of patients who received any systemic therapy decreased with increasing age. While 45.6% (742) of patients less than 55 years of age received systemic therapy, only 7.6% (188) of patients 80 or older received any form of systemic therapy. In addition, the median survival amongst the group was shorter amongst the elderly (3.2 months in those ≥80 years of age) than amongst younger age groups (5.5 and 4.8 months, respectively, for patients <55 and 55–59 years of age).

### 3.2. Total Costs Across Age Groups

**Figure 3:**
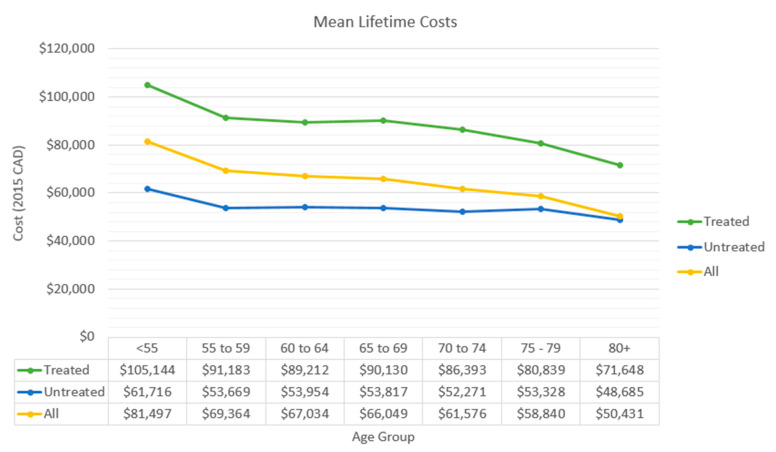
Average total lifetime costs incurred by cancer patients who received systemic therapy, those who did not receive systemic therapy, and the total population.

After diagnosis of cancer, younger patients incurred a higher average lifetime total cost than older patients (Figure 3). This was especially the case amongst patients who received systemic therapy, with the youngest group incurring the highest cost (CAD 105,144 in ≤55 years old) and the oldest group incurring the lowest cost (CAD 71,648 in >80 years old) (Figure 3).

**Figure 4:**
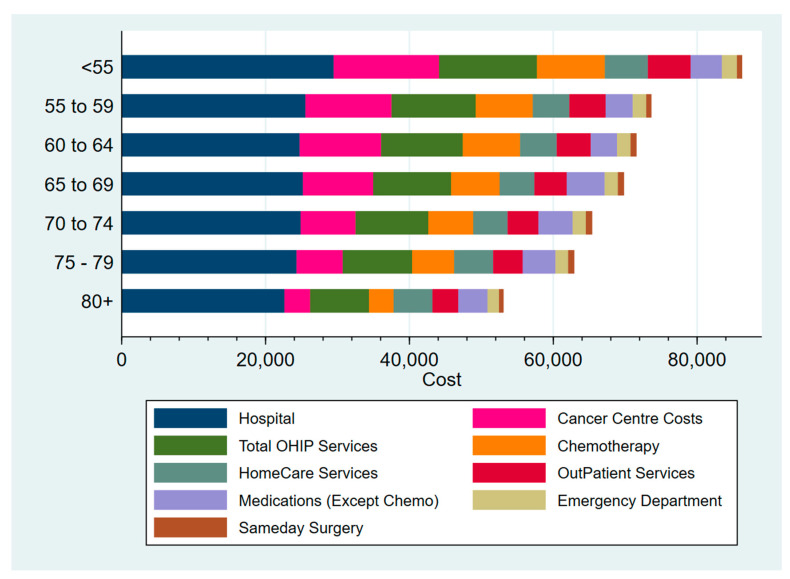
Average lifetime cancer-care cost by age and category of healthcare costs, amongst patients who received systemic therapy.

Prior to the era of immunotherapy, major contributors to costs in the younger groups appeared to be hospitalizations, cancer-care costs (cancer center and chemotherapy costs), and other associated health services billings (Figure 4). While hospitalization and other associated health services billings remained constant, cancer-care costs appeared to decrease with age (Figure 4). Amongst all patients, the highest categories of healthcare spending were hospitalizations (278%), outpatient cancer care not including medication costs (21%), and other outpatient care visits (16%). Cancer medications and home care each contributed 8% to the total cost of care (Table S1). Qualitative examination of the medians of the costs revealed a pattern that was similar to the average costs.

### 3.3. Average Monthly CAC by Phase of Care Across Age Groups

**Figure 5:**
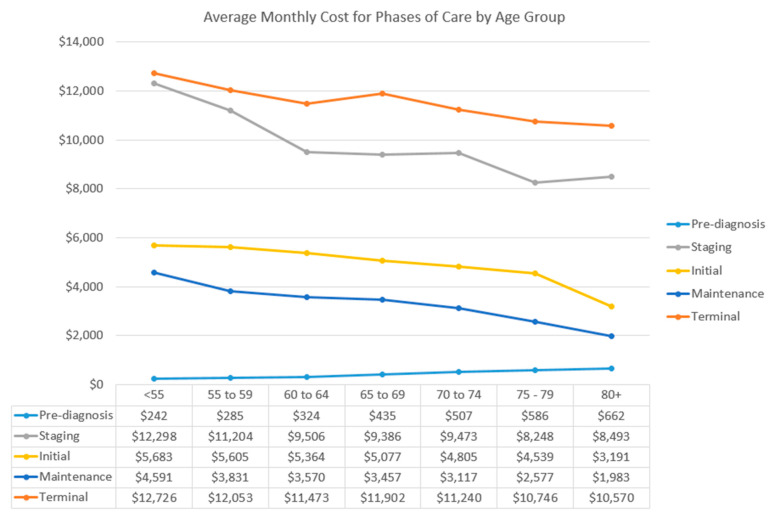
Average monthly costs by phase of care across age groups.

Figure 5 shows the average monthly costs by phase of care across age groups in the pre- and post-diagnosis phases. Post-diagnosis phase costs are average monthly CACs. Details of the figure, including monthly costs by phase of care, can be found in Supplementary Table S2. Regardless of age group, staging and end-of-life phases incurred higher costs compared with initial and continuing phases of care. In addition, costs of all post-diagnosis phases appeared to decrease with increasing age. Costs increased by age in the pre-diagnosis phase. The average pre-diagnosis costs were much less than those of the post-diagnosis phases (Figure 5).

### 3.4. Costs During Pre-Diagnosis Phase

Prior to cancer diagnosis, increasing age led to increased baseline costs to the healthcare system (Table 2). For example, prior to the diagnosis of cancer, patients aged ≥80 at diagnosis cost 5.45 times more than patients <55 years of age (univariate adjusted relative cost 5.45, 95% C.I. 4.95–6.01, *p* < 0.01). This pattern remained significant (multivariate adjusted relative cost 4.93, 95% C.I. 4.46–5.44, *p* < 0.01) after adjusting for gender, income, treatment, year of diagnosis, and whether patients’ primary residence was rural or urban (Table 2). Detailed multivariate linear regression analysis is found in Supplementary Table S3.

### 3.5. Costs During Post-Diagnosis Phases: Staging, Initial, Continuing, and End-of-Life Phases of Care

After the diagnosis of advanced NSCLC, unlike the pre-diagnosis phase, elderly patients incurred significantly less cost to the healthcare system than that the youngest age group in all four phases of care (Table 3). This remained significant after adjusting for potential confounders including gender, income, comorbidities, year of diagnosis, living rurally, and whether systemic therapy was administered (Table 3 and Table S3). Thus, after cancer diagnosis, increasing age was associated with decreasing CACs to the healthcare system.

### 3.6. Factors Affecting Costs

Some factors contributed similarly to cost throughout the phases of care (Supplementary Table S3). For example, having a more recent year of diagnosis was associated with higher costs of care throughout the staging, initial, and continuing phases of care. In the initial and continuing phases, the costs of patients diagnosed in 2013 were 1.41 (95% C.I. 1.27–1.57, *p* < 0.01) and 1.72 (95% C.I. 1.47–2.02, *p* < 0.01) times those of patients diagnosed in 2008, respectively. In the pre-diagnosis phase of care, there were no significant trends in cost of care from 2008 to 2013.

Other factors contributed differentially across different post-diagnosis phases of care (Supplementary Table S3). Receiving chemotherapy contributed to higher costs than not receiving chemotherapy in the initial and continuing phases of care. For example, patients who received chemotherapy incurred 2.11 times the costs of patients who did not receive any chemotherapy during the continuing phase (95% C.I. 1.90–2.33, *p* < 0.01). Opposite trends were seen during the pre-diagnosis and end-of-life phase of care. Patients who received chemotherapy cost 0.77 (95% C.I. 0.72–0.81, *p* < 0.01) and 0.74 (95% C.I. 0.72–0.79, *p* < 0.01) times those who did not receive any chemotherapy in the pre-diagnosis and end-of-life phases of care, respectively While socioeconomic status (SES, as represented by income quintiles) was not associated with changes in cost across any of the post-diagnosis phases of care, higher SES was associated with lower costs of care in the pre-diagnosis phase).

In the staging and end-of-life phase of care, living in a rural community was associated with slightly lower cost than living in an urban community (staging adjusted relative cost 0.87 95% C.I. 0.79–0.97, *p* < 0.01; end-of-life adjusted relative cost 0.93 95% C.I. 0.89–0.96, *p* < 0.00). Being male was associated with slightly higher costs at the end-of-life phase of care (adjusted relative cost 1.06 95% C.I. 1.04–1.10, *p* < 0.01).

## 4. Discussion

Using a provincial population-based database, our study was able to assess the real-world CACs for advanced NSCLC patients as they navigated various phases of care, including baseline costs prior to cancer diagnosis. Using this, we found that older age appeared to be associated with differential costs to the public healthcare system prior to, and after, diagnosis of advanced NSCLC. Prior to cancer diagnosis, older age was associated with higher costs to the healthcare system, this pattern was reversed when assessing CACs after cancer diagnosis. The higher costs incurred by younger patients after cancer diagnosis persisted even after adjusting for potential confounders across different post-diagnosis phases of care.

Similar to our current study, studies from other countries, including those from the US, UK, Greece, and Spain, found that cancer-care costs in older patient cohorts were lower than those of younger patient [5,6,7,10,11,12]. In China, a population-level study by Zhu et al., appeared to show a normal distribution, where cost of NSCLC care was highest in the 55–64-year-old age group, and lower in both younger and elderly (75+) age groups [13]. Previous studies have also suggested varying healthcare resource use for NSCLC patients across countries [14]. This could also be due to variations in costs captured amongst countries. An understanding of the underlying cause of these regional differences in costs across age groups may help identify if these differences are driven by economic or clinical variations.

In assessing costs associated with various phases of care, Kaye et al. demonstrated through the SEER-Medicare data, that cost of cancer care was highest in the initial and end-of-life phases, and lower in the continuing phase of care [12]. A similar pattern of a “U”-shaped cost distribution was shown in a review of 30 studies published in the US between 2013 and 2017 [15]. This previously described “U”-shaped cost distribution was demonstrated across all age groups in our study as well [16]. The lower costs in the continuing phase could be due to the relatively preserved quality of life during this phase, when patients are on treatment and not requiring significant interventions such as multiple hospitalizations. Although this is the current pattern of the CACs, the uptake of costly therapies such as immunotherapy and targeted therapies may increase the cost of therapy and shift the pattern of healthcare utilization for advanced NSCLC patients.

Although not comparing across phases of care, Gentili et al. found more than double the mean per-patient total costs following the introduction of immunotherapy (EUR 19,301 vs. EUR 7804) [17]. There, the main driver of higher costs was for treatment, which comprised 66.3% of the total costs. Similarly, population-level studies already suggest an increase in the cost of therapies with the introduction of targeted therapy and immunotherapy in second- or subsequent-line treatments [18,19]. Although the cost of treatment only comprised 8.1% of the total costs in our study, there was a statistically significant increase in cost of care in the more recent years of diagnosis that may have been driven by increased treatment costs [17]. We predict that more prevalent utilization of immunotherapy in the front-line setting is likely to disproportionately increase costs during the continuing phase of care. However, with improved treatment options, we are seeing a longer time on treatment and longer survival, thus both the initial and continuing phases are likely to be longer in duration, and costs may be offset by improved quality of life and lower hospitalization rates [20].

When assessing net monthly costs, our study was on par with prior studies in a similar period, although net costs were higher in the end-of-life phase. For example, our 65–69-years-old cohort cost on average CAD 11,902/month in 2015 CAD or USD 9760 in 2015 USD (using purchasing power parity calculations) compared to USD 7281/month (2015 USD) in one US study [21]. This may have been due to variations in the definition of the end-of-life phase. Banegas et al. defined the end-of-life phase as the last 12 months of life, compared to the 6 months for our study. This suggests an increased peak in costs during the last 6 months of life as opposed to last 12 months, which is more in keeping with findings from previous studies and the life expectancy and natural history of advanced NSCLC patients [22,23,24,25]. It is also possible that the Ontario database, a publicly funded system, captures a different subset of the costs incurred [12,23]. In addition, there may also be differences in the organization of end-of-life care that results in different costs in this phase of care. This highlights a need to standardize data collection and classification of time periods of phases of care for future studies.

Fairly unique to our study, was the capture of a spectrum of age-related healthcare costs immediately prior to cancer diagnosis. We found, like other population-level studies, that elderly patients at baseline incurred higher healthcare costs to the system [26,27]. Thus, the lower CAC for elderly patients supports previous findings that there is a decrease in uptake in cancer treatments after elderly patients are diagnosed with cancer [2,3,28]. Furthermore, our study showed that cancer center costs also disproportionately decreased with age, which suggests that elderly patients may never meet an oncologist to discuss treatment options. This finding is in keeping with previous publications by Dawe et al. [2]. This may be inappropriate, as previous studies have shown that elderly patients stand to gain similar benefits from treatment in quality of life and survival compared to their younger counterparts [29,30,31]. These observations may reflect undertreatment, whether due to provider bias, comorbidities, or a lower referral rate to medical oncologists for consideration of systemic therapy. Although there exists the possibility that lower end-of-life costs in elderly patients may also stem from patient preference for less intensive care, elderly patients still deserve access to specialists and the ability to choose. Thus, for clinicians, the possibility of a systemic bias in provision of care should be considered when assessing elderly patients for systemic therapy.

This study had relevance in budgetary impact analyses, which need to account for changes in anticipated demographics and treatment patterns. Although we anticipated an aging demographic with a higher proportion of elderly patients, this group appeared to incur lower costs on the healthcare system than younger patients. However, with the increasing use of targeted therapies and immune check-point inhibitors, which tend to have fewer toxicities and result in better quality of life compared with traditional chemotherapy, this pattern may no longer hold as there may be an increase in the proportion of elderly patients seeking and being provided with systemic therapy. When the cost of immunotherapy is estimated to be over 10 times the cost of traditional chemotherapy, the cost of care attributable to therapies will likely become the dominant contributor in CACs. This, in turn, will also increase health care utilization and costs associated with management of adverse events associated with these systemic therapies.

## 5. Limitations

This study was limited to capturing direct costs incurred by the healthcare system, as indirect costs are not captured within IC-ES. Thus, the patient perspective could not be assessed using the data within this study. Furthermore, data on oral systemic therapy was not available for patients under 65 years of age. In our cohort, 35% of patients were under the age of 65 and may, therefore, have incomplete data on the use of oral systemic therapy. This limitation likely contributed to an underestimation of the CACs in this subgroup. In spite of this potential bias, elderly patients still had much lower CACs compared to the <65 age groups. Given that oral systemic therapy is used in a relatively small proportion of patients, the overall impact on the cost estimate was likely minimal.

Our categorization of costs, albeit following the methodology developed by colleagues in health economics, had an impact on estimates of cost for each category. For example, those with short survival contributed more to the end-of-life phase and less to the other phases of care and the continuing phase costs only represented those people who did well on treatment and lived longer. This potential bias did not change the proportional drop in costs of care with increasing age, seen throughout all post-diagnosis phases of care.

In addition, our study focused on deceased patients with stage IV NSCLC, to create a more homogeneous cohort of patients and capture CACs across the complete cancer trajectory. This approach minimized confounding from unrelated healthcare utilization that can occur in earlier-stage or long-term survivor populations. While this design improved attribution of cancer-related costs, it may have limited generalizability to survivors. At the time of the analysis, however, most patients (96%) within our cohort were deceased, reducing the risk of survivor bias.

Finally, this study was retrospective in nature and ended at the start of the incorporation of immune check-point inhibitors, which, depending on pattern of usage, may significantly affect the costs incurred by age group. However, this study remains relevant as the baseline trends established across age groups serve as an important benchmark for evaluating future studies with newer treatments.

## 6. Conclusions

Amongst patients diagnosed with advanced NSCLC, increasing age was associated with lower CACs across all phases of care following diagnosis, despite being associated with significantly higher costs prior to diagnosis. Previous studies have highlighted the potential disparities in the uptake of cancer treatments for elderly patients, and our findings further underscore the reduced healthcare utilization for elderly patients after diagnosis of advanced NSCLC. We look forward to assessing whether this trend shifts in the era of newer, less toxic treatments. A thorough analysis of healthcare utilization by age is needed to determine if this reflects an appropriate adjustment in intensity of care, or if it represents missed opportunities to optimize cancer care for elderly patients.

## Figures and Tables

**Table 1:** Baseline demographics of stage IV NSCLC patients across age groups.

	Age Group	<55	55–59	60–64	65–69	70–74	75–79	80+	Overall
Gender	Female (n)	819	720	950	1031	1100	951	1130	6701
	%	50	49	46	44	46	43	46	
	Male (n)	810	750	1139	1341	1302	1270	1342	7954
	%	50	51	55	57	54	57	54	
Morphology	Adenoca (n)	944	754	1056	1170	1119	1039	1135	7211
	%	58	51	51	49	47	47	46	
	SCC (n)	170	188	299	392	443	430	467	2387
	%	10	13	14	17	18	19	19	
	NSCLC-NOS (n)	439	477	639	717	743	682	794	4491
	%	27	32	31	30	31	31	32	
	Other (n)	76	51	95	93	97	70	76	558
	%	5	4	5	4	4	3	3	
Lived rurally	Yes (n)	229	231	344	397	392	285	222	2100
	%	14	16	17	17	16	13	9	
	No (n)	1399	1238	1743	1972	2010	1934	2249	12,545
	%	86	84	84	83	84	87	91	
Income quintile	1st (lowest) (n)	378	356	483	559	501	466	509	3252
	%	23	24	23	24	21	21	21	
	2nd (n)	359	325	462	531	537	504	539	3257
	%	22	22	22	23	22	23	22	
	3rd (n)	342	294	387	455	520	432	489	2919
	%	21	20	19	19	22	19	20	
	4th (n)	287	280	389	422	446	430	461	2735
	%	18	19	19	18	19	19	19	
	5th (highest) (n)	256	208	355	371	387	381	464	2422
	%	16	14	17	16	16	17	19	
	Missing (n)	7	7	13	14	11	8	10	70
	%	0	0	1	1	0	0	0	
Chemo	Yes (n)	742	615	775	799	655	445	188	4219
	%	46	42	37	34	27	20	8	
	No (n)	887	855	1314	1573	1747	1776	2284	10,436
	%	54	58	63	66	73	80	92	
OS	Median (months)	5.5	4.8	4.9	4.2	4.1	3.9	3.2	4.2
	25% (months)	2.4	2.2	2.1	1.8	1.8	1.7	1.4	1.8
	75% (months)	13.1	11.3	11.0	10.3	9.4	9.3	7.7	10.1

Adenoca—adenocarcinoma, SCC—squamous cell carcinoma, NSCLC-NOS—non-small-cell lung cancer—not otherwise specified, chemo—chemotherapy, OS—overall survival.

**Table 2:** Multivariate linear regression analysis of mean monthly costs in the pre-diagnosis phase.

Age Group	Adjusted Relative Cost	95% C.I. Lower Bound	95% C.I. Higher Bound	*p* Value
<55	Reference	Reference	Reference	Reference
55–59	1.24	1.11	1.38	<0.01
60–64	1.42	1.28	1.57	
65–69	2.43	2.21	2.69	
70–74	3.35	3.03	3.69	
75–79	4.12	3.72	4.55	
80+	4.93	4.46	5.44	

**Table 3:** Multivariate analysis of CACs in the post-diagnosis phases.

Phase of Care	Age Group	Adjusted Relative Cost	95% C.I. Lower Bound	95% C.I. Higher Bound	*p* Value
Staging (n = 5428)	<55	Reference	Reference	Reference	Reference
	55–59	0.83	0.72	0.96	<0.01
	60–64	0.69	0.60	0.79	
	65–69	0.66	0.58	0.76	
	70–74	0.67	0.59	0.77	
	75–79	0.54	0.47	0.63	
	80+	0.52	0.45	0.61	
Initial (n = 47,812)	<55	Reference	Reference	Reference	Reference
	55–59	1.02	0.90	0.15	<0.01
	60–64	0.89	0.80	0.99	
	65–69	0.91	0.82	1.02	
	70–74	0.86	0.77	0.96	
	75–79	0.82	0.73	0.92	
	80+	0.61	0.54	0.69	
Continuing (n = 2461)	<55	Reference	Reference	Reference	Reference
	55–59	0.93	0.78	1.11	<0.01
	60–64	0.85	0.73	1.00	
	65–69	0.81	0.69	0.95	
	70–74	0.74	0.63	0.87	
	75–79	0.70	0.59	0.84	
	80+	0.59	0.49	0.71	
End of life (n = 14,513)	<55	Reference	Reference	Reference	Reference
	55–59	0.94	0.88	0.99	<0.01
	60–64	0.88	0.83	0.93	
	65–69	0.89	0.84	0.93	
	70–74	0.80	0.76	0.85	
	75–79	0.73	0.69	0.77	
	80+	0.67	0.64	0.71	

## Data Availability

The data presented in this study are available in this article (and Supplementary Materials).

## Supplementary Materials

The following supporting information can be downloaded at: https://www.mdpi.com/article/10.3390/curroncol32060346/s1, Table S1: Percentage contribution and mean costs of various categories of care amongst patients who received treatment (n = 4219); Table S2: Average monthly CAC by phase of care across age groups (2015 CAD$); Table S3: Multivariate linear regression analysis adjusting for clinical factors associated with cost differential across various phases of care.
