# Supplementary material for: Cost Disparities with Age in the Treatment of Advanced Non-Small-Cell Lung Cancer (NSCLC) in Ontario, Canada
Source: Curr Oncol. 2025 Jun 12;32(6):346. doi: 10.3390/curroncol32060346 (PMC12191781; doi:10.3390/curroncol32060346)
Supplement: Supplementary file 1 [file curroncol-32-00346-s001.zip › curroncol-3648421-supplementary.pdf]

Supplementary Materials

Table S1. Percentage contribution and mean costs of various categories of care amongst patients who received treatment (n = 4219).

Category of Care	Mean Costs	% of Total Cost of Care
Hospitalization	$24,836	27.7%
Cancer Centre Services	$19,117	21.3%
OHIP Services	$14,538	16.2%
Chemotherapy	$7,290	8.1%
Home Care Services	$7,394	8.2%
Outpatient Services	$6,338	7.1%
Medications (except for chemotherapy)	$6,971	7.8%
Emergency Department Services	$2,203	2.5%
Sameday Surgery Services	$1,025	1.1%
Total Average Cost of Care (per patient)	$89,710	100.0%

OHIP – Ontario Health Insurance Plan

Table S2. Average monthly CAC by phase of care across age groups (2015 CAD$).

Staging Phase	N	Mean cost	Std. Dev.
<55	766	$12,297.99	$12,883.33
55 - 59	619	$11,204.33	$13,614.69
60 - 64	891	$9,506.04	$10,250.64
65 - 69	925	$9,386.03	$12,354.12
70 - 74	887	$9,473.33	$11,088.53
74 - 79	817	$8,247.50	$10,594.32
80+	765	$8,493.45	$10,441.69
Initial Phase	N	Mean cost	Std. Dev.
<55	712	$5,683.02	$5,035.85
55 - 59	553	$5,605.37	$5,894.64
60 - 64	817	$5,363.79	$5,447.61
65 - 69	813	$5,076.75	$4,222.65
70 - 74	790	$4,805.27	$5,190.06
74 - 79	724	$4,539.37	$6,364.57
80+	652	$3,191.33	$4,027.30
Continuing Phase	N	Mean cost	Std. Dev.
<55	407	$4,590.52	$6,824.53
55 - 59	297	$3,830.82	$3,070.95
60 - 64	432	$3,570.15	$3,388.40
65 - 69	450	$3,457.39	$3,111.05
70 - 74	398	$3,117.01	$3,637.56
74 - 79	363	$2,576.96	$3,175.25
80+	324	$1,983.38	$3,669.15
Terminal Phase	N	Mean cost	Std. Dev.
<55	1626	$12,726.48	$11,956.38
55 - 59	1467	$12,052.50	$11,536.65
60 - 64	2088	$11,473.03	$10,315.17
65 - 69	2371	$11,902.43	$10,439.37
70 - 74	2402	$11,239.83	$10,630.25
74 - 79	2221	$10,745.69	$10,139.19
80+	2472	$10,570.10	$9,814.75

Table S3. Multivariate linear regression analysis adjusting for clinical factors associated with cost differential across various phases of care.

Phase of care	Factor	Categories	Adjusted Relative Cost	95% C.I. Lower Bound	95% C.I. Higher Bound	P value
Pre-diagnosis (n=13950)	Gender	Female	Reference	Reference	Reference	Reference
		Male	1.01	0.97	1.07	0.57
	Year of diagnosis	2008	Reference	Reference	Reference	Reference
		2009	0.95	0.87	1.04	>0.05
		2010	1.01	0.93	1.11	
		2011	0.97	0.89	1.06	
		2012	0.94	0.86	1.03	
		2013	0.93	0.88	1.05	
	Geography	Urban	Reference	Reference	Reference	Reference
		Rural	0.96	0.90	1.04	0.31
	Chemotherapy	No	Reference	Reference	Reference	Reference
		Yes	0.77	0.72	0.81	<0.01

	Factor	Categories	Adjusted Relative Cost	95% C.I. Lower Bound	95% C.I. Higher Bound	P value
Staging (n=5310)	Gender	Female	Reference	Reference	Reference	Reference
		Male	1.02	0.95	1.10	0.52
	Year of diagnosis	2008	Reference	Reference	Reference	Reference
		2009	1.29	1.13	1.48	<0.01
		2010	1.09	0.96	1.24	
		2011	1.27	1.12	1.45	
		2012	1.35	1.19	1.54	
		2013	1.43	1.25	1.63	
	Geography	Urban	Reference	Reference	Reference	Reference
		Rural	0.87	0.79	0.97	<0.01
	Chemotherapy	No	Reference	Reference	Reference	Reference
		Yes	0.93	0.86	1.01	0.09

	Factor	Categories	Adjusted Relative Cost	95% C.I. Lower Bound	95% C.I. Higher Bound	P value
Initial (n=4701)	Gender	Female	Reference	Reference	Reference	Reference
		Male	0.97	0.91	1.03	0.25
	Year of diagnosis	2008	Reference	Reference	Reference	Reference
		2009	1.16	1.04	1.30	<0.01
		2010	1.30	1.17	1.45	
		2011	1.25	1.12	1.39	
		2012	1.38	1.24	1.54	
		2013	1.41	1.27	1.57	
	Geography	Urban	Reference	Reference	Reference	Reference
		Rural	0.98	0.90	1.08	0.70
	Chemotherapy	No	Reference	Reference	Reference	Reference
		Yes	1.48	1.39	1.58	<0.01

	Factor	Categories	Adjusted Relative Cost	95% C.I. Lower Bound	95% C.I. Higher Bound	P value
Continuing (n=2405)	Gender	Female	Reference	Reference	Reference	Reference
		Male	0.94	0.86	1.03	0.21
	Year of diagnosis	2008	Reference	Reference	Reference	Reference
		2009	1.19	1.01	0.40	<0.01
		2010	1.22	1.05	1.43	
		2011	1.40	1.20	1.63	
		2012	1.74	1.48	2.04	
		2013	1.72	1.47	2.02	
	Geography	Urban	Reference	Reference	Reference	Reference
		Rural	0.88	0.77	1.00	0.05
	Chemotherapy	No	Reference	Reference	Reference	Reference
		Yes	2.11	1.90	2.33	<0.01

	Factor	Categories	Adjusted Relative Cost	95% C.I. Lower Bound	95% C.I. Higher Bound	P value

	Gender	Female	Reference	Reference	Reference	Reference
		Male	1.06	1.04	1.10	<0.01
	Year of diagnosis	2008	Reference	Reference	Reference	Reference
		2009	1.07	1.02	1.13	
		2010	1.03	0.99	1.09	
End of Life (n=14191)		2011	1.10	1.05	1.15	<0.01
		2012	1.09	1.04	1.15	
		2013	1.13	1.08	1.19	
	Geography	Urban	Reference	Reference	Reference	Reference
		Rural	0.93	0.89	0.96	<0.01
	Chemotherapy	No	Reference	Reference	Reference	Reference
		Yes	0.74	0.72	0.77	<0.01
